# Loss/gain-induced ultrathin antireflection coatings

**DOI:** 10.1038/srep28681

**Published:** 2016-06-28

**Authors:** Jie Luo, Sucheng Li, Bo Hou, Yun Lai

**Affiliations:** 1 College of Physics, Optoelectronics and Energy & Collaborative Innovation Center of Suzhou Nano Science and Technology, Soochow University, Suzhou 215006, China

## Abstract

Tradional antireflection coatings composed of dielectric layers usually require the thickness to be larger than quarter wavelength. Here, we demonstrate that materials with permittivity or permeability dominated by imaginary parts, i.e. lossy or gain media, can realize non-resonant antireflection coatings in deep sub-wavelength scale. Interestingly, while the reflected waves are eliminated as in traditional dielectric antireflection coatings, the transmitted waves can be enhanced or reduced, depending on whether gain or lossy media are applied, respectively. We provide a unified theory for the design of such ultrathin antireflection coatings, showing that under different polarizations and incident angles, different types of ultrathin coatings should be applied. Especially, under transverse magnetic polarization, the requirement shows a switch between gain and lossy media at Brewster angle. As a proof of principle, by using conductive films as a special type of lossy antireflection coatings, we experimentally demonstrate the suppression of Fabry-Pérot resonances in a broad frequency range for microwaves. This valuable functionality can be applied to remove undesired resonant effects, such as the frequency-dependent side lobes induced by resonances in dielectric coverings of antennas. Our work provides a guide for the design of ultrathin antireflection coatings as well as their applications in broadband reflectionless devices.

Antireflection coatings (ARCs) have been designed to reduce the reflection of electromagnetic waves on the interface of two dielectrics with different refractive indexes. The purpose of ARCs is not only to increase transmission, but also to eliminate the reflected waves so as to avoid hazards such as ghost images in optical systems, optical pollution due to blaze on glass curtain walls, etc. Single layered dielectric ARCs of quarter wavelength thicknesses have been successfully applied in optics. Such ARCs only works for limited ranges of frequencies and incident angles. Graded-refractive-index ARCs, with impedance gradually changing, have been designed to expand the ranges of working frequencies and incident angles^1^,^2^,^3^,^4^,^5^,^6^,^7^,^8^. However, the geometry sizes of graded-refractive-index ARCs are even larger, usually comparable to the wavelength of incident waves.

Recent advances in metamaterials^9^,^10^,^11^,^12^ and metasurfaces^13^,^14^ made it possible to realize ultrathin ARCs by using microstructures of sub-wavelength thickness^15^,^16^,^17^. In addition, high-index materials with Mie resonances^18^, surface plasmon resonances^19^,^20^,^21^ and guided resonances^22^ have also been demonstrated to realize ARCs. However, due to their resonance nature, most of these approaches were limited in narrow frequency ranges.

On the other hand, it is known that a conductive film (or resistive sheet) can operate as impedance matching layer between two dielectric media in microwave regime^23^,^24^,^25^. Recently, conductive films are also proposed as ultrathin ARCs in the terahertz regime^26^,^27^,^28^,^29^,^30^,^31^,^32^,^33^,^34^,^35^. The functionality of such ARCs is broadband and asymmetric, i.e. reflections can be eliminated when the waves are incident from a material with larger dielectric constant to another material with lower dielectric constant, but not vice versa^26^. Despite the special case of conductive films, however, it is not clear if there exist other types of non-resonant ultrathin ARCs, as well as their working conditions.

In this work, by imposing the conditions of constant tangential electric or magnetic fields in an ultrathin layer, we have developed a unified theory for non-resonant ultrathin ARCs. We find that materials with permittivity or permeability dominated by imaginary parts, i.e. lossy or gain media, is necessary to realize these ultrathin ARCs. Interestingly, while the reflected waves are eliminated as in traditional dielectric ARCs, the transmitted waves can be enhanced or reduced, depending on whether gain or lossy media are applied. Besides conductive films, our theory predicts some other types of non-resonant ultrathin ARCs, such as ARCs composed of strong gain media as the opponent of conductive films, and ARCs composed of zero-index media (ZIM) with tiny loss or gain. In particular, under transverse magnetic (TM) polarization, we find that the Brewster angle is a critical angle on which the requirement of ARCs is switched from lossy media to gain media, or vice versa. Our work vastly expands the types of non-resonant ARCs and also systematically classifies their working conditions.

A significant advantage of such non-resonant ARCs is the feasibility of achieving broadband functionality. Such a property makes the suppression of Fabry-Pérot (FP) resonances in a broad frequency range possible. Here, by using a type of ultrathin conductive films which have been developed extensively in optoelectronics^36^,^37^, we have experimentally verified the suppression of FP resonances in dielectric slabs, for both normal incidence and oblique incidence, in a wide frequency regime from 5 GHz to 17 GHz. We further demonstrate that by attaching such conductive films to the dielectric covering of an antenna, the frequency-dependent side lobes induced by resonances in the dielectric covering can be significantly reduced, leading to a wideband stable radiation signature.

We would like to distinguish the functionality of ARCs with that of perfect absorbers in the case of lossy media. For perfect absorbers, the purpose is to maximize absorption and therefore, both reflection and transmission are eliminated. However, for lossy ARCs proposed here, the reflection is eliminated, and there is still a considerable amount of transmitted waves through the ARCs even though the transmission is reduced. Moreover, the original refractive behavior of the interface between two dielectric media is maintained in the transmitted waves. Such transmitted waves can transport information and have important applications such as lensing and signal processing. As we shall demonstrate later, the conditions for ARCs are very different from those for perfect absorption.

## Results and Discussions

### Unified theory for ultrathin antireflection coatings

We consider that an electromagnetic plane wave is incident from lossless dielectric medium 1 with relative permittivity *ε*_1_ into lossless dielectric medium 2 with relative permittivity *ε*_2_ at incident angle *θ*. Then the reflection coefficient can be easily calculated as,


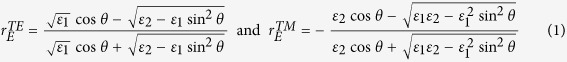


for transverse electric (TE) polarization (with electric field in the *y* direction) and TM polarization (with magnetic field in the *y* direction), respectively. The reflection coefficients are defined as the ratios of reflected and incident electric fields. For incident angles under the critical angle associated with total reflection, 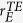 and 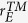 are always real numbers.

**Figure 1:**
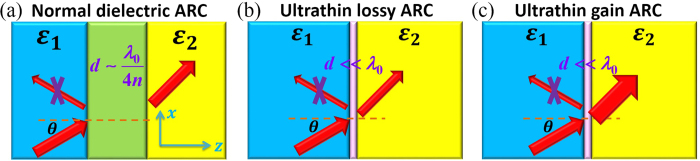
Schematics of different kinds of ARCs. Illustration of three types of ARCs by using (**a**) a quarter-wave dielectric layer, (**b**) an ultrathin layer of lossy media, and (**c**) an ultrathin layer of gain media. The thicknesses of the ultrathin layers are in deep subwavelength scale.

To eliminate the reflection, we place a uniform layer between the dielectric media 1 and 2, as illustrated in Fig. 1(a–c). Traditionally, the additional layer is chosen as a quarter-wave dielectric layer with a relative permittivity of 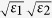 and a minimal thickness of 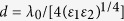, as illustrated in Fig. 1(a). *λ*_0_ is the wavelength in free space. Evidently, the thickness of the dielectric layer is comparable to quarter wavelength.

Now, we assume that ultrathin layers with thickness in deep wavelength scale can also exhibit antireflection effects, i.e. become ARCs, as illustrated in Fig. 1(b,c), and theoretically investigate the conditions of such ultrathin ARCs. In order to avoid any FP resonances inside the layer, we assume that the phase change in such an ultrathin layer is negligible, i.e.,


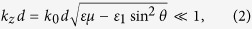


where *k*_*z*_ is the *z* component of wave vectors. *ε*(*μ*) and *d* are the relative permittivity (permeability) and thickness of the layer, respectively. *k*_0_ (=2*π*/*λ*_0_) is the wave number in free space.

With the limit of Eq. (2), the resonance effect of the ultrathin layer is much reduced. As a result, we consider the cases that either tangential electric or magnetic fields are almost constant across the layer. However, if both the tangential electric and magnetic fields are constant, the ultrathin layer is totally transparent and cannot operate as ARCs. Therefore, we should consider the cases that only the tangential electric or magnetic fields are constant across the film.

**Figure 2:**
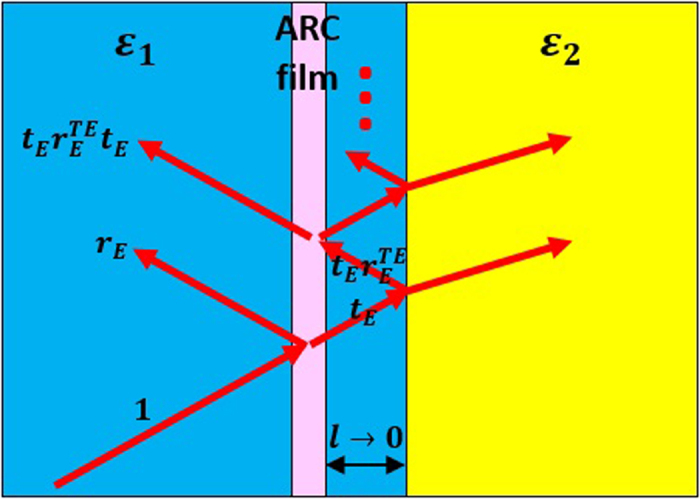
Illustration of the derivation of the antireflection condition for the case of constant tangential electric fields.

Firstly, we consider the case of constant tangential electric fields across the film. That is, the reflection and transmission coefficients of the ARC film in the background medium 1 satisfy 1 + *r*_*E*_ = *t*_*E*_ and 1 + *r*_*H*_ ≠ *t*_*H*_, where *r*_*E*_ (*r*_*H*_) and *t*_*E*_ (*t*_*H*_) are, respectively, the reflection and transmission coefficients defined on the ratio of electric (magnetic) fields. In Fig. 2, we assume that a dielectric medium 1 slab with thickness tending to be zero (i.e., *l* → 0) is placed between the ARC film and the dielectric medium 2. Thus, the total reflection coefficient of the whole system can be calculated as the sum of multiple reflections:


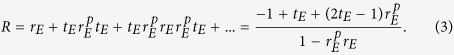


where 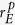 denotes 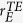 (*p* = *TE*) or 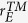 (*p* = *TM*) for TE or TM polarization. And they can be obtained from Eq. (1).

The condition of antireflection is *R* = 0, which yields 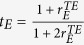 under the condition of 1 + *r*_*E*_ = *t*_*E*_. By combining Eq. (2) and the expression of *t*_*E*_ obtained by using transfer matrix method^38^, the antireflection condition can be written as,


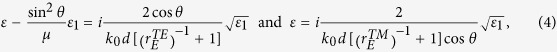


for TE and TM polarizations, respectively. Equation (4) describes the required parameters of the ultrathin ARC film. We note that similar method has been applied to obtain the parameters for ultrathin perfect absorption^39^. For perfect absorption, however, medium 2 is chosen to be either perfect electric (magnetic) conductor, metals, or photonic band gap materials that can block electromagnetic waves. The obtained requirements for perfect absorption are thus generally different from those for ARCs, as presented in this paper. One typical example is that for ARCs, there is a switch of medium requirement at the Brewster angle for TM polarization. For perfect absorption, however, there is no such a switch of requirement.

Under different circumstances, Eq. (4) can be simplified to some simple forms. Here, as a simple summary, we present the obtained ARC types from Eq. (4) in Table 1. We will discuss them in details in the next section.

Secondly, for the case with constant tangential magnetic fields across the film, the required parameters of the ultrathin ARC film can be similarly derived as,


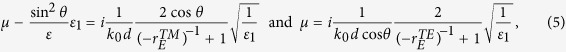


for TM and TE polarizations, respectively.

Similarly, Eq. (5) can also be simplified to give several ARC types under different circumstances, as is presented in Table 2.

We note that both Eqs. (4) and (5) give the exact solutions of ultrathin ARCs. The difference between them lies in the constant tangential electric field [Eq. (4)] or constant tangential magnetic field [Eq. (5)] across the layer.

### Discussions of materials for ultrathin antireflection coatings

For simplicity, here we only consider the case of constant tangential electric field. In the following, we will discuss different solutions derived from Eq. (4), which are listed in Table 1. As for the case of constant tangential magnetic field, analysis can be carried out similarly to obtain Table 2.

### ARCs composed of lossy and gain media

For an ultrathin layer, we have *k*_0_*d* ≪ 1. Therefore, if the permeability *μ* of the ARC is normal (e.g. *μ* = 1 as in most nonmagnetic materials), and the incident angle *θ* is not too large, the permittivity *ε* of the ARC is required to have a large imaginary part. For TE polarization, it can be seen from Eq. (4) that when 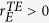, *ε* has a positive imaginary part and the ARC is composed of lossy media. From Eq. (1), it can be seen that the condition of 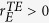 is satisfied for any incident angle if the dielectric media 1 and 2 satisfy *ε*_1_ > *ε*_2_. On the other hand, if *ε*_1_ < *ε*_2_, we have 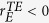 for any incident angle. When 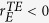, from Eq. (4), it can be seen that *ε* has a negative imaginary part and thus the ARC is composed of gain media. This conclusion is a little counter-intuitive, as gain media usually lead to strong radiation like lasing.

In fact, the gain media solution for ARCs can be understood from the time-reversal point of view. The lossy media solution requires *ε*_1_ > *ε*_2_, i.e. incidence from the medium with a higher refractive index. When time is reversed, the situation is changed into incidence from the medium with a lower refractive index. At the same time, lossy medium is changed into gain medium. The lossy or gain ARCs only work in an asymmetric manner, as the media do not fulfill the conditions of *ε* = ε^*^ and *μ* = μ^*^. This is different from previous quarter-wave dielectric ARCs, which is symmetric.

**Figure 3:**
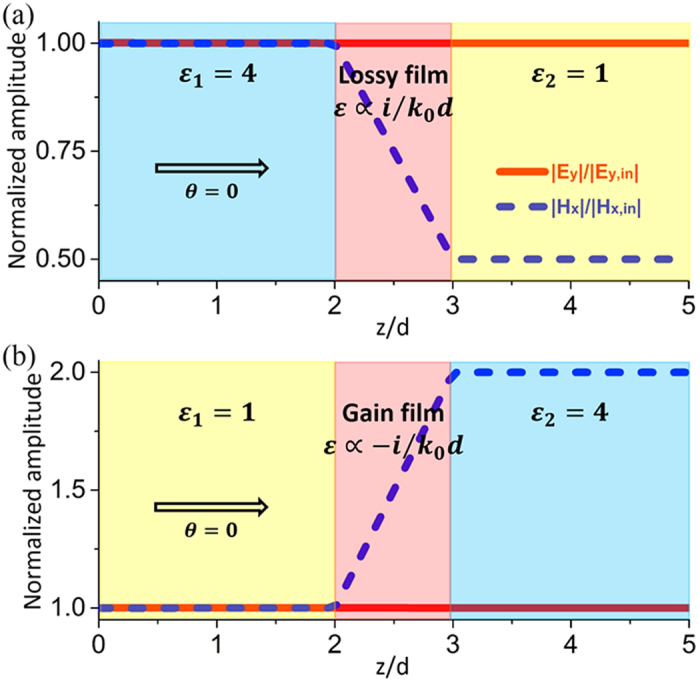
ARCs composed of lossy or gain media. Simulated normalized amplitudes of electric and magnetic fields inside the ultrathin nonmagnetic ARC with (**a**) *ε* = 79.58*i* ∝ *i*/*k*_0_*d* and (**b**) *ε* = −79.58*i* ∝ −*i*/*k*_0_*d* under normal incidence. The thickness of the ARC is *d* = *λ*_0_/500. The relative permittivities of the dielectric media 1 and 2 are, respectively, (**a**) *ε*_1_ = 4 and *ε*_2_ = 1, (**b**) *ε*_1_ = 1 and *ε*_2_ = 4.

To verify the antireflection effects for both lossy and gain ARCs, we have performed simulations by using finite element software COMSOL Multiphysics. In Fig. 3(a), we consider TE polarized waves normally incident from a dielectric medium with *ε*_1_ = 4 into free space with *ε*_2_ = 1. Based on Eq. (4), we find that a lossy nonmagnetic ultrathin layer with *ε* = 79.58*i* ∝ *i*/*k*_0_*d* and *d* = *λ*_0_/500 is capable of eliminating the reflections. In Fig. 3(a), we present the normalized amplitudes of electric field |*E*_*y*_|/|*E*_*y*,*in*_| (red solid lines) and magnetic field |*H*_*x*_|/|*H*_*x*,*in*_| (blue dashed lines), where *E*_*y*,*in*_ and *H*_*x*,*in*_ are the *y*-component of incident electric field and *x*-component of incident magnetic field, respectively. The unity fields in dielectric medium 1 indicate that there are no reflections. In Fig. 3(b), we consider the reversed situation of *ε*_1_ = 1 and *ε*_2_ = 4. It is demonstrated that an ARC made of gain media with *ε* = −79.58*i* ∝ −*i*/*k*_0_*d* and *d* = *λ*_0_/500 can realize antireflection. The electric fields are constant inside the ARC layer, which is in consistent with our theory. However, the magnetic fields are reduced or enhanced, respectively, in the cases of lossy or gain ARCs. This indicates that the transmitted flux can be reduced or enhanced.

In practice, the lossy ARC can be easily realized by using conductive films, as we shall discuss in details later. The gain ARC implies a negative conductance. Recently, theories based on such gain media with negative conductance have been proposed to realize negative refraction and cloaking, in which some realization schemes have been proposed^40^,^41^.

### ARCs composed of ZIM for oblique incidence

There is another unique solution of the ARC for TE polarization in Eq. (4). When the permittivity *ε* of the ARC is normal (e.g. of the order of low index dielectrics like air or glass), then the permeability *μ* of the ARC is required to be approaching zero for oblique incidence. More specifically, a particular approximate solution can be obtained as


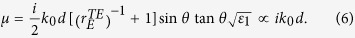


In this case, interestingly, the material of the ARC is a type of ZIM with permeability |*μ*| ≪ 1. We note that the permeability is still almost purely imaginary, i.e. the real part of permeability should be much smaller than the imaginary part. However, this is the typical case for most ZIM in practice^42^,^43^,^44^,^45^,^46^,^47^,^48^,^49^,^50^. Amazingly, such ARC made of ZIM only requires a tiny amount of loss or gain for the realization of antireflection. When the thickness of the ZIM film approaches zero, i.e., *d* → 0, we have |*μ*| → 0.

ZIM operating as ARCs is also counter-intuitive, because it is known that ZIM generally reflect almost all the incident waves under oblique incidence^42^,^43^,^44^. However, when the parameters of ZIM is not extreme, ZIM can allow electromagnetic waves to penetrate inside^45^ and even operate as perfect absorbers^39^,^46^,^47^,^48^,^49^,^50^. Here, for the first time, we show that ZIM can also operate as ultrathin ARCs.

**Figure 4:**
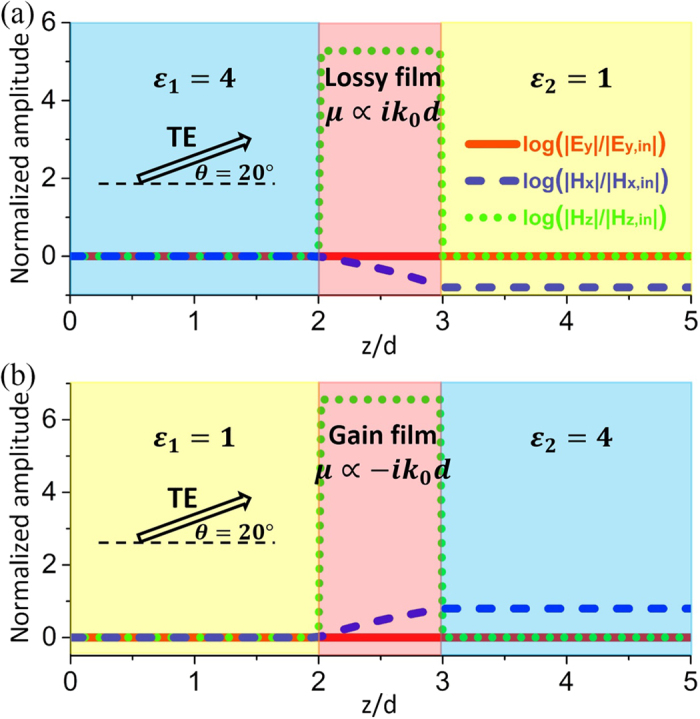
ARCs composed of ZIM for oblique incidence. Simulated normalized amplitudes of electric and magnetic fields inside the ultrathin ARC composed of ZIM with (**a**) *ε* = 1, *μ* = 0.0051*i* ∝ *ik*_0_*d* and (**b**) *ε* = 1, *μ* = −0.0014*i* ∝ −*ik*_0_*d* under oblique incidence with *θ* = 20°. The thickness of the ARC is *d* = *λ*_0_/500. The relative permittivities of the dielectric media 1 and 2 are, respectively, (**a**) *ε*_1_ = 4 and *ε*_2_ = 1, (**b**) *ε*_1_ = 1 and *ε*_2_ = 4.

In Fig. 4(a,b), we have performed numerical simulations to verify antireflection effects of the ARCs composed of ZIM. In Fig. 4(a), the parameters of dielectric media 1 and 2 are *ε*_1_ = 4 and *ε*_2_ = 1, respectively. TE polarized waves are incident from the left side with an incident angle of *θ* = 20°. From Eq. (4), it is found that the ARC can be composed of lossy ZIM layers with *ε* = 1, *μ* = 0.0051*i* ∝ *ik*_0_*d* and *d* = *λ*_0_/500. From the distribution of normalized amplitudes of electric field |*E*_*y*_|/|*E*_*y*,*in*_| (red solid lines) and magnetic field |*H*_*x*_|/|*H*_*x*,*in*_| (blue dashed lines) and |*H*_*z*_|/|*H*_*z*,*in*_| (green dotted lines), we can see that there are no reflections in dielectric medium 1. *E*_*y*,*in*_, *H*_*x*,*in*_ and *H*_*z*,*in*_ are the *y*-component of incident electric fields, *x*-component and *z*-component of incident magnetic fields, respectively. In Fig. 4(b), we consider the other case: the parameters of dielectric media 1 and 2 are *ε*_1_ = 1 and *ε*_2_ = 4, respectively. Under the same incident angle, the antireflection effect is clearly demonstrated by using the ARC composed of gain ZIM films with *ε* = 1, *μ* = −0.0014*i* ∝ −*ik*_0_*d* and *d* = *λ*_0_/500. In Fig. 4(a,b), we also notice that electric fields are constant, while the tangential magnetic fields are linearly decreased or increased. In addition, the normal component of magnetic fields are enhanced greatly inside the ARC due to the near-zero permeability.

In practice, lossy ZIM can be readily fabricated by using various approaches, including semiconductors^51^,^52^, metamaterials^53^,^54^,^55^, meta-dielectric composites^56^,^57^, waveguides within cut-off frequency^58^,^59^ and photonic crystals with Dirac dispersion^60^,^61^, etc. Although the requirement of loss or gain is tiny in this case, the reduction or enhancement in the transmitted waves is exactly the same as the previous case with large loss and gain. Since the electric fields are constant across the ARC, the magnetic fields of transmitted waves are actually only determined by the impedance mismatch between dielectric media 1 and 2.

### TM polarization and transition at the Brewster angle

For TM polarization, we can derive the conditions from the second equation in Eq. (4). Similarly, we find that 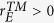 corresponds to the ARCs made of lossy media, while 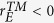 corresponds to the ARCs made of gain media.

However, the sign of 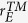 is not only simply determined by the ratio of *ε*_1_/*ε*_2_, but also changes with the increase of incident angles. From Eq. (1), we find that Brewster angle *θ*_*B*_ is a critical angle on which the sign of 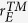 switches from positive (negative) to negative (positive) for the case with *ε*_1_ > *ε*_2_ (*ε*_1_ < *ε*_2_). Therefore, the case of TM polarization is very different from the case of TE polarization. Both lossy and gain media can operate as the ARCs for both cases of *ε*_1_ > *ε*_2_ and *ε*_1_ < *ε*_2_, but there is a switch of the lossy and gain media at the Brewster angle *θ*_*B*_.

**Figure 5:**
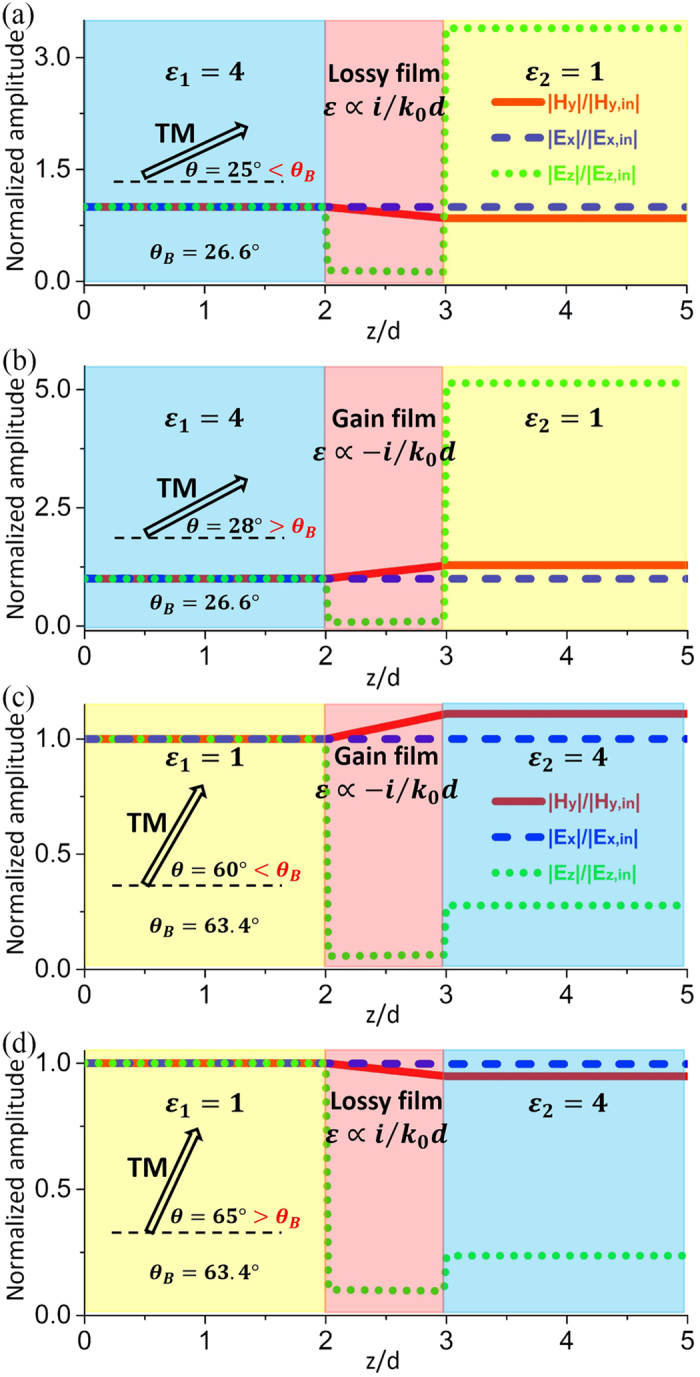
Transition at the Brewster angle for TM polarization. Simulated normalized amplitudes of electric and magnetic fields inside the ultrathin nonmagnetic ARC with (**a**) *ε* = 26.70*i* ∝ *i*/*k*_0_*d* for *θ* = 25°, (**b**) *ε* = −51.03*i* ∝ −*i*/*k*_0_*d* for *θ* = 28°, (**c**) *ε* = −17.41*i* ∝ −*i*/*k*_0_*d* for *θ* = 60°, and (**d**) *ε* = 9.76*i* ∝ *i*/*k*_0_*d* for *θ* = 65°. The thickness of the ARC is *d* = *λ*_0_/500. The relative permittivities of the dielectric media 1 and 2 are, respectively, (**a,b**) *ε*_1_ = 4 and *ε*_2_ = 1, (**c,d**) *ε*_1_ = 1 and *ε*_2_ = 4.

To clearly demonstrate this unique feature, we have performed numerical simulations for TM polarization, as shown in Fig. 5(a–d). Firstly, we consider the case of *ε*_1_ = 4 and *ε*_2_ = 1. In this case, the Brewster angle is about *θ*_*B*_ = 26.6°. When the incident angle is *θ* = 25° (smaller than the Brewster angle), the nonmagnetic ARC with a thickness of *d* = *λ*_0_/500 should be composed of lossy media with *ε* = 26.70*i* ∝ *i*/*k*_0_*d*, as shown in Fig. 5(a). However, if the incident angle is *θ* = 28° (larger than the Brewster angle), the nonmagnetic ARC should be composed of gain media with *ε* = −17.41*i* ∝ −*i*/*k*_0_*d*, as shown in Fig. 5(b). From the distribution of normalized amplitudes of magnetic field |*H*_*y*_|/|*H*_*y*,*in*_| (red solid lines), electric field |*E*_*x*_|/|*E*_*x*,*in*_| (blue dashed lines) and |*E*_*z*_|/|*E*_*z*,*in*_| (green dotted lines) in Fig. 5(a,b), it is seen that there are no reflection waves in dielectric medium 1. *H*_*y*,*in*_, *E*_*x*,*in*_ and *E*_*z*,*in*_ are the *y*-component of incident magnetic fields, *x*-component and *z*-component of incident electric fields, respectively. In addition, we see that the tangential electric fields are constants, while the tangential magnetic fields vary inside the ARC, which is in consistent with our theory.

Secondly, we consider the case with *ε*_1_ = 1 and *ε*_2_ = 4. In this case, the Brewster angle is about *θ*_*B*_ = 63.4°. From Fig. 5(c,d), it is seen that if the incident angle is *θ* = 60° (or *θ* = 65°), the nonmagnetic ARC with a thickness of *d* = *λ*_0_/500 should be composed of gain (or lossy) media with *ε* = −17.41*i* ∝ −*i*/*k*_0_*d* (or *ε* = 9.76*i* ∝ *i*/*k*_0_*d*). The distributions of normalized amplitudes of electric and magnetic fields also indicate that there are no reflected waves in dielectric medium 1.

To the best of our knowledge, the findings shown above have not been reported in previous literature. Actually, most of previous works^23^,^24^,^25^,^26^,^27^,^28^,^29^,^30^,^31^,^32^,^33^,^34^,^35^ have only considered the case of normal incidence, in which ARCs composed of lossy media only work for the cases of *ε*_1_ > *ε*_2_, but not for *ε*_1_ < *ε*_2_. Here, for the first time, we show exceptions and interesting possibilities for TM polarization.

### Broadband lossy ARCs composed of conductive films

In the above discussions, we have shown that for normal incidence, the required permittivity satisfies *ε* ∝ ± *i*/*k*_0_*d*, which indicates that the permittivity of the ARCs has a large pure imaginary part, when *k*_0_*d *→ 0. From Eq. (4), we can see that *ε* ∝ ± *i*/*k*_0_*d* is always true for TM polarization. For TE polarization, the result is the same for nonmagnetic ARCs with *μ* = 1, except for some particular cases, such as *ε*_1_ ≫ 1, or *ε*_1_ ≈ *ε*_2_, or *θ* → 90°.

In particular, the solution of lossy media with *ε* ∝ *i*/*k*_0_*d* is unique because it implies that the ultrathin ARCs can be realized by using conductive films in an extremely broad frequency range. As we known, the relative permittivity of a conductive film with a conductivity *σ*_0_ is described as 
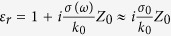
, where *Z*_0_ is the impedance of free space and *σ*_0_ is the conductivity at zero frequency (DC case)^39^,^62^,^63^. Combined with the antireflection condition in Eq. (4), we find that the conductive film operates as a ARC if the sheet resistance, *R*_*s*_, defined as 1/(*σ*_0_*d*), satisfies,


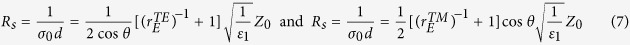


for TE and TM polarizations, respectively.

**Figure 6:**
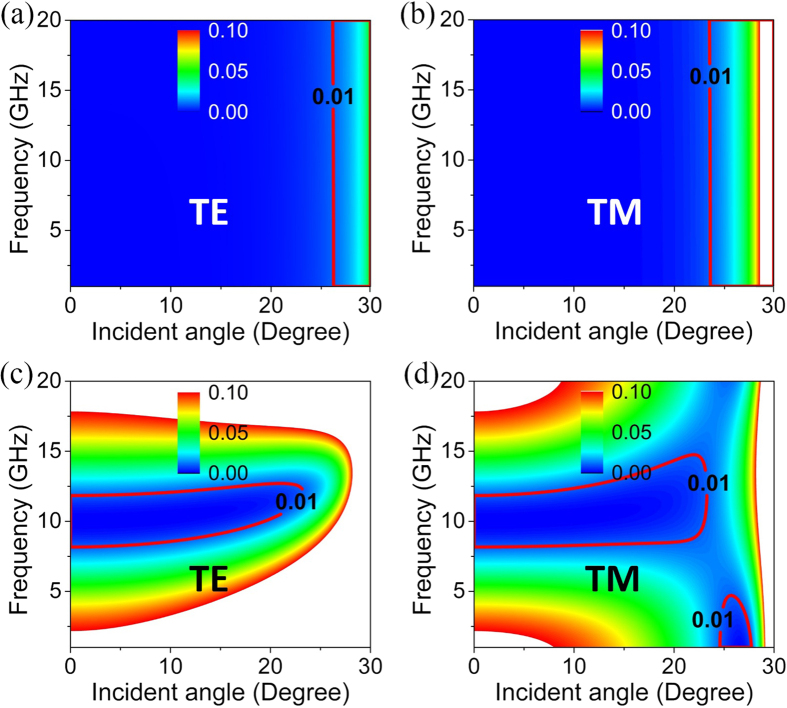
Broadband lossy ARCs composed of conductive films. Reflectance as the function of the incident angle and the working frequency for (**a**,**c**) TE and (**b**,**d**) TM polarizations. The ARCs composed of (**a,b**) a conductive film with *R*_*s*_ = *Z*_0_ and *d* = 0.06 mm, (**c,d**) a quarter-wave dielectric film with *ε* = 2 and *d* = 5.3 mm. The relative permittivities of the dielectric media 1 and 2 are *ε*_1_ = 4 and *ε*_2_ = 1, respectively.

Apparently, the required sheet resistance *R*_*s*_ is independent of frequency, implying that this is an extremely broadband ARC. In principle, the operating frequency can extend from the quasi-static regime to THz regime. The ARC functionality only fails when the frequency increases to a regime where the approximation of constant conductivity is inaccurate. To verify such an interesting feature, we plot the reflectance (defined as the ratio of reflected and incident energy flux) as the function of the incident angle and operating frequency in Fig. 6(a,b) for TE and TM polarizations, respectively. The parameters of dielectric media 1 and 2 are *ε*_1_ = 4 and *ε*_2_ = 1, respectively. And the conductive ARC is characterized by *R*_*s*_ = *Z*_0_ and *d* = 0.06 mm. With such an ultrathin conductive ARC, the reflectance for both TE and TM polarizations can be drastically reduced in a broad band and a wide angle range. We note that the maximal incident angle is a bit smaller than 30°, which is the critical angle of total reflection.

For comparison, we consider a quarter-wave dielectric ARC with *ε* = 2 and *d* = 5.3 mm. Here, we note that the thickness is considerably large as the wavelengths are relatively large at microwave frequencies. Figure 6(c,d) present the reflectance with respect to incident angles and operating frequency for TE and TM polarizations, respectively. Apparently, the quarter-wave ARC can only work in a narrow range of working frequency and incident angle.

In summary, ARCs composed of conductive films exhibit extremely broadband, wide-angle, and polarization-insensitive characteristics, which are absent in quarter-wave dielectric ARCs. Such lossy ARCs may be especially useful in some applications which put the elimination of reflection rather than the increase of transmission first. Although there exist transmission losses due to absorption, there are still a considerable amount of transmitted waves, which can carry information and be used for applications such as lensing and signal processing.

### Experimental verification of suppression of FP resonances in a broad frequency band

**Figure 7:**
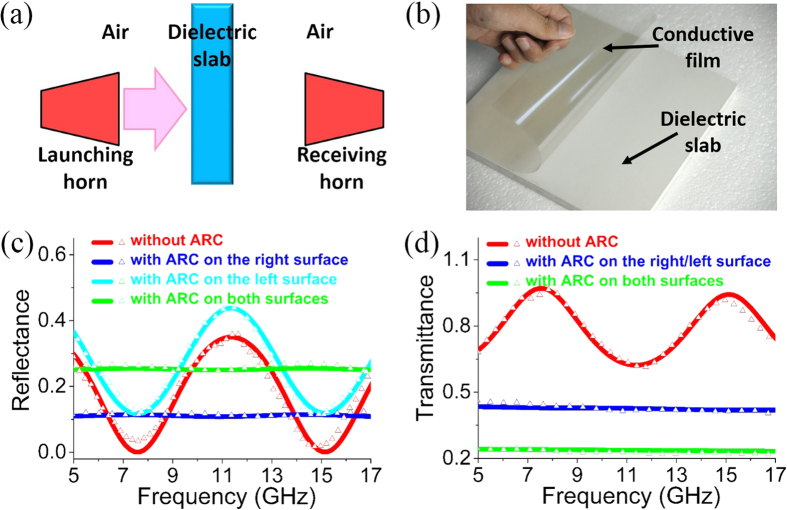
Experiments in free spaces for normal incidence. (**a**) Illustration of the experimental setup in free space. (**b**) Photo of the experimental sample. Simulated (solid lines) and measured (triangular symbols) (**c**) reflectance and (**d**) transmittance under normal incidence. The relevant parameters of the dielectric slab are *ε*_1_ = 4 + 0.03*i* and *d*_1_ = 9.9 mm. The conductive film has a sheet resistance of *R*_*s*_ = 370 Ω and a conducting layer thickness of 2.6 μm.

**Figure 8:**
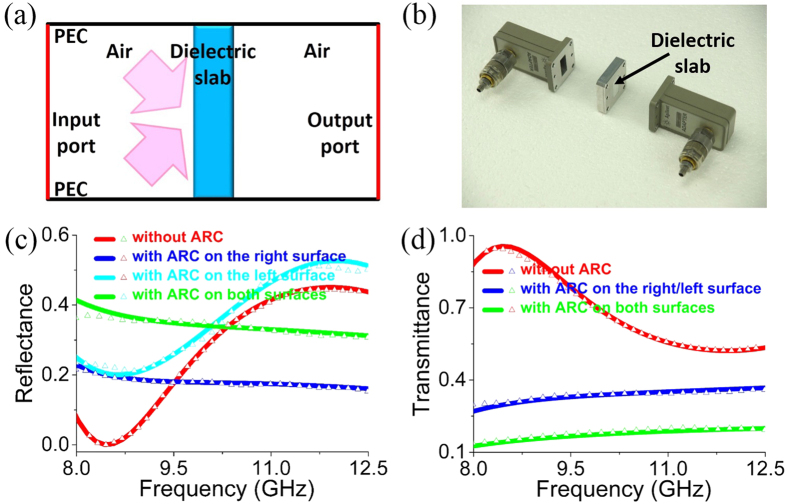
Experiments in waveguides for oblique incidence. (**a**) Illustration and (**b**) photo of the experimental setup of rectangular waveguides. Simulated (solid lines) and measured (triangular symbols) (**c**) reflectance and (**d**) transmittance in the waveguide with TE_10_ modes. The relevant parameters of the dielectric slab are *ε*_1_ = 4 + 0.03*i* and *d*_1_ = 9.6 *mm*. The conductive film has a sheet resistance of *R*_*s*_ = 370 Ω and a conducting layer thickness of 2.6 μm.

As we known, when a dielectric slab is placed in air, FP resonances may occur in the dielectric slab, leading to frequency-dependent reflection and transmission spectra. Interestingly, if the right or/and left surface of the dielectric slab is coated with an ARC composed of conductive films with appropriate sheet resistance, FP resonances can be eliminated in a broadband frequency range and also for a wide range of incident angles. To verify this interesting feature, we have performed microwave experiments with experimental setup illustrated in Figs 7(a) and 8(a).

Firstly, we perform the normal incidence experiment in free space. Figure 7(a,b) show the illustration of the experimental setup and the photo of the sample, respectively. In experiment, two horn antennas are employed to launch and receive the microwave with frequency covering 5–17 GHz. A dielectric slab characterized by a relative permittivity *ε*_1_ = 4 + 0.03*i* and a thickness *d*_1_ = 9.9 mm is placed in microwave passage.

Figure 7(c,d) show the simulated (solid lines) and measured (triangular symbols) reflectance and transmittance, respectively. The red lines in Fig. 7(c,d) denote the reflectance and transmittance spectra, respectively, for the dielectric slab without ARCs. Apparently, the reflectance and transmittance oscillate when the frequency increases, indicating the existence of FP resonances. Interestingly, when the right surface of the dielectric slab is coated with an ultrathin conductive film (which is a commercial transparent electrode^36^,^37^) with a sheet resistance of *R*_*s*_ = 370 Ω (which is very close to ideal value *Z*_0_) and a conducting layer thickness of 2.6 μm, the reflectance [blue lines in Fig. 7(c)] and transmittance [blue lines in Fig. 7(d)] for the waves incident from the left are almost constant in the measuring frequency range. This indicates that the FP resonances are eliminated in a wide frequency band, as the result of antireflection effects of the conductive film. However, if the left surface is coated with the conductive film, the light blue lines in Fig. 7(c) show that the oscillation of reflection exists, which is caused by the interference of the first two orders of reflections. The transmittance is the same as that for the case with the conductive film coated on the right surface. Finally, we coat both the left and right surfaces of the dielectric slab with the conductive ARC. And the reflectance and transmittance spectra are denoted by the green lines in Fig. 7(c,d), respectively. The frequency-independent reflectance and transmittance demonstrate that the FP resonances in the dielectric slab are eliminated in a broad frequency band.

Secondly, we perform the oblique incidence experiment by utilizing a WR90 rectangular waveguide (transversal size 22.86 × 10.16 mm^2^), inside which TE_10_ modes are excited. Figure 8(a,b) show the illustration and photo of experimental setup, respectively. In an alternative view, TE_10_ modes can be decomposed into two plane wave propagating at the same oblique angle *θ* = cos^−1^(*β*/*k*_0_) with *β* being the propagation constant inside the waveguide. To make sure the single-mode operation, the measuring frequency is below 13 GHz, which is determined by the TE_20_ cutoff frequency. As the operating frequency changes from 8 GHz to 12.5 GHz, the related angle decreases from 55.1° to 31.7°. Therefore, we could check whether the conductive film is efficient or not for a wide range of incident angles.

The relevant parameters of the dielectric slab are *ε*_1_ = 4 + 0.03*i* and *d*_1_ = 9.6 mm. When the dielectric slab is uncoated with the ARC, both reflectance and transmittance oscillate as functions of frequency, as shown by the red solid lines (simulated data) and triangular symbols (measured data) in Fig. 8(c,d). However, when the right surface of the dielectric slab is coated with a conductive film with a sheet resistance of 370 Ω, the oscillation of the reflectance and transmittance for the left incident wave is dramatically suppressed, as displayed by the blue solid lines (simulated data) and triangular symbols (measured data) in Fig. 8(c,d). We note that the reflectance (transmittance) is slightly decreased (increased) as the frequency increases, as the result of the decreased incident angle. Similar to the case of normal incidence, the reflectance for the case with ARC coated on the left surface depends on the operating frequency, as shown by the light blue solid lines (simulated data) and triangular symbols (measured data) in Fig. 8(c). We have also coated both surfaces of the dielectric slab, and the resultant reflectance and transmittance are plotted in Fig. 8(c,d) by green lines (simulated data) and symbols (measured data). The smooth data prove the suppression of FP resonances inside the dielectric slab in a wide range of incident angles.

### An example of application in antenna design

**Figure 9:**
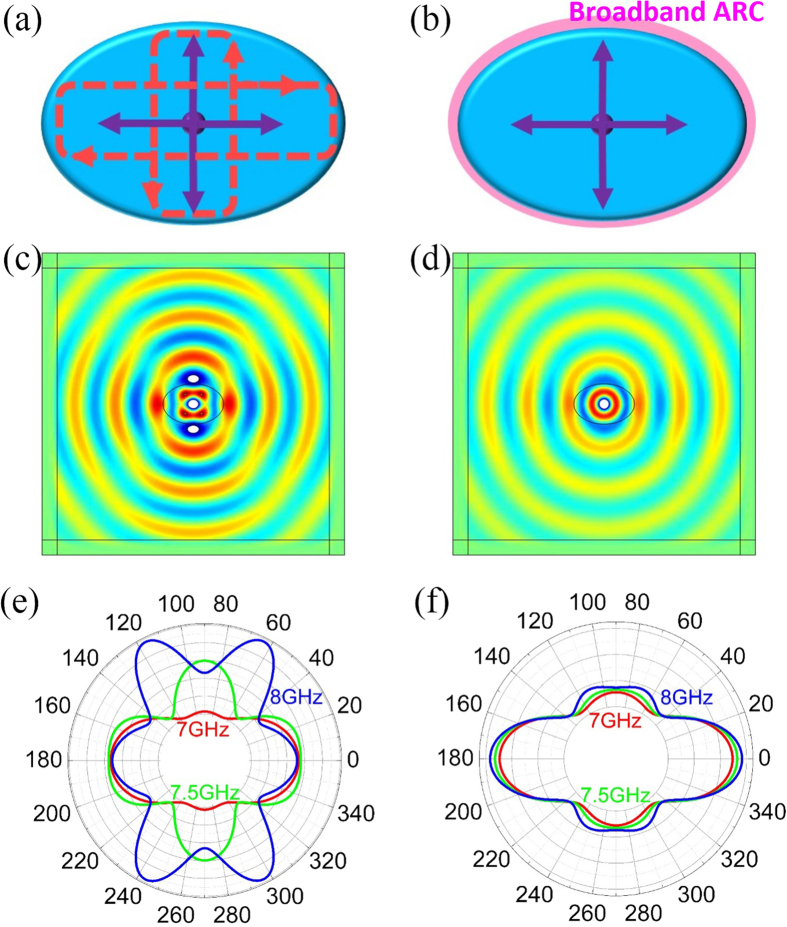
An example of application in antenna design. Schematic of the electric monopole antenna with an elliptically shaped dielectric shell when the conductive ARC is (**a**) uncoated and (**b**) coated. Snapshot of electric fields produced by the antenna at the 7.5 GHz (**c**) without and (**d**) with the conductive ARC. Far-field patterns of electric fields at 7 GHz, 7.5 GHz and 8 GHz (**e**) without and (**f**) with the conductive ARC.

In the following, we demonstrate an application of the suppression of FP resonances: removing the frequency-dependent side lobes in the radiation signature of an antenna which are induced by resonances in its dielectric covering. Dielectric coverings have been widely applied to protect antenna against extreme environment. Figure 9(a) displays an electric antenna excited by out-of-plane current of 1A (as a monopole in the plane), which is coated by an elliptically shaped dielectric shell with a relative permittivity of 4. Since the impedance of the dielectric shell and air is mismatched, reflections occur at the interface of the two media, leading to resonances inside the shell, as illustrated by the red dashed lines in Fig. 9(a). As a result, the radiation signature of the antenna is added with complicated side lobes, which is strongly dependent on frequency. Figure 9(c) depicts the electric field distribution at 7.5 GHz, showing apparent resonances. Moreover, the far-field patterns of electric fields are plotted in Fig. 9(e) at 7 GHz (red lines), 7.5 GHz (green lines) and 8 GHz (blue lines), showing side lobes with frequency-dependent behaviors. However, if a conductive ARC with *R*_*s*_ = *Z*_0_ is coated on the outer surface of the dielectric shell [shown in Fig. 9(b)], the internal reflections will be removed. As expected from Fig. 9(d), the standard monopole radiation in the dielectric shell is observed at 7.5 GHz. From the far-field patterns of 7 GHz (red lines), 7.5 GHz (green lines) and 8 GHz (blue lines) displayed in Fig. 9(f), we can see that the radiation signature turns out to be almost frequency-independent. Thereby, we can stabilize the radiation patterns of antennas by applying the broadband and ultrathin conductive ARC.

## Conclusion

In conclusion, we have derived the unified formulas for the design of loss/gain-induced ultrathin ARCs with constant tangential electric or magnetic fields. Our theory shows that various types of loss/gain media with parameters dominated by imaginary parts can realize antireflection effects, however, under different conditions of polarizations and incident angles. For TM polarization, the Brewster angle is a critical angle at which the requirement changes from gain media to lossy media, or vice versa. Furthermore, we point out that such loss/gain-induced ultrathin ARCs have important applications. By using ultrathin conductive films, we have experimentally demonstrated the suppression of FP resonances in a broad frequency range and a wide angle range for microwaves. Such characteristics can be applied to stabilize the radiation signatures of antennas with dielectric coverings. Our work opens possibilities for the design of novel electromagnetic devices such as broadband non-reflective lenses and other non-resonant instruments.

## Additional Information

**How to cite this article**: Luo, J. *et al*. Loss/gain-induced ultrathin antireflection coatings. *Sci. Rep.* **6**, 28681; doi: 10.1038/srep28681 (2016).

## Figures and Tables

**Table 1:** Summary of required media for ARCs based on Eq. (4).

	TE	TM	
		*θ* < *θB*	*θ* > *θB*
*ε*_1_ > *ε*_2_	Lossy media *ε* ∝ *i/k*_0_*d or μ* ∝ *ik*_0_*d or other parameters satisfying* Eq. (4)	Lossy media *ε* ∝ *i*/*k*0*d*	Gain media *ε* ∝ −*i*/*k*0*d*
*ε*_1_ < *ε*_2_	Gain media *ε* ∝ *i/k*_0_*d or μ* ∝ −*ik*_0_*d or other parameters satisfying* Eq. (4)	Gain media *ε* ∝ −*i*/*k*0*d*	Lossy media *ε* ∝ *i*/*k*0*d*

**Table 2:** Summary of required media for ARCs based on Eq. (5).

	TE	TM	
		*θ* < *θB*	*θ* > *θB*
*ε*_1_ > *ε*_2_	Gain media *μ* ∝ −*i*/*k*0*d*	Gain media *ε* ∝ *ik*_0_*d or μ* ∝ −*i/k*_0_*d or other parameters satisfying* Eq. (5)	Lossy media *ε* ∝ *ik*_0_*d or μ* ∝ −*i/k*_0_*d or other parameters satisfying* Eq. (5)
*ε*_1_ < *ε*_2_	Lossy media *μ* ∝ *i*/*k*0*d*	Lossy media *ε* ∝ *ik*_0_*d or μ* ∝ *i/k*_0_*d or other parameters satisfying* Eq. (5)	Gain media *ε* ∝ −*ik*_0_*d or μ* ∝ −*i/k*_0_*d or other parameters satisfying* Eq. (5)
